# Experimental and Natural Infections of Goats with Severe Fever with Thrombocytopenia Syndrome Virus: Evidence for Ticks as Viral Vector

**DOI:** 10.1371/journal.pntd.0004092

**Published:** 2015-10-20

**Authors:** Yongjun Jiao, Xian Qi, Dapeng Liu, Xiaoyan Zeng, Yewu Han, Xiling Guo, Zhiyang Shi, Hua Wang, Minghao Zhou

**Affiliations:** 1 Institute of Pathogenic Microbiology, Jiangsu Provincial Center for Disease Prevention and Control, Key Laboratory of Enteric Pathogenic Microbiology, Ministry Health, Nanjing, China; 2 Xuyi County Center for Disease Prevention and Control, Huai-an, China; The Kenya Medical Research Institute, KENYA

## Abstract

**Background:**

Severe fever with thrombocytopenia syndrome virus (SFTSV), the causative agent for the fatal life-threatening infectious disease, severe fever with thrombocytopenia syndrome (SFTS), was first identified in the central and eastern regions of China. Although the viral RNA was detected in free-living and parasitic ticks, the vector for SFTSV remains unsettled.

**Methodology/Principal Findings:**

Firstly, an experimental infection study in goats was conducted in a bio-safety level-2 (BSL-2) facility to investigate virus transmission between animals. The results showed that infected animals did not shed virus to the outside through respiratory or digestive tract route, and the control animals did not get infected. Then, a natural infection study was carried out in the SFTSV endemic region. A cohort of naïve goats was used as sentinel animals in the study site. A variety of daily samples including goat sera, ticks and mosquitoes were collected for viral RNA and antibody (from serum only) detection, and virus isolation. We detected viral RNA from free-living and parasitic ticks rather than mosquitoes, and from goats after ticks’ infestation. We also observed sero-conversion in all members of the animal cohort subsequently. The S segment sequences of the two recovered viral isolates from one infected goat and its parasitic ticks showed a 100% homology at the nucleic acid level.

**Conclusions/Significance:**

In our natural infection study, close contact between goats does not appear to transmit SFTSV, however, the naïve animals were infected after ticks’ infestation and two viral isolates derived from an infected goat and its parasitic ticks shared 100% of sequence identity. These data demonstrate that the etiologic agent for goat cohort’s natural infection comes from environmental factors. Of these, ticks, especially the predominant species *Haemaphysalis longicornis*, probably act as vector for this pathogen. The findings in this study may help local health authorities formulate and focus preventive measures to contain this infection.

## Author Summary

Severe fever with thrombocytopenia syndrome virus (SFTSV), a newly identified bunyavirus, has been found to circulate in mainland China, South Korea, and Japan since 2009. This virus is the etiologic agent for an emerging fatal hemorrhagic fever, severe fever with thrombocytopenia syndrome (SFTS) with high fatality. Although ticks have been implicated as the primary host vector indicated by epidemiological surveys, their role in transmitting this virus to the susceptible hosts, including humans, has not been validated. In this study, we conducted experimental and natural infections of goats with SFTSV to explore the role of ticks for this pathogen’s transmission. In the experimental infection study, we have not found any viral transmission within the cohort by close contact between animals. However, in the natural infection study, every member of a naïve goat cohort was observed to get infected sequentially when they were farmed in a SFTSV-endemic site. We detected viral RNA from free-living and parasitic ticks rather than mosquitoes, and from goats after ticks’ infestation. We also observed sero-conversion in all members of the animal cohort subsequently. More importantly, in the natural infection study, two virus strains isolated from one infected goat and its parasitic ticks showed identical S segment sequences of the viral genome. All these findings indicate that ticks, especially the dominant species *Haemaphysalis longicornis*, probably act as viral vector for this emerging pathogen, SFTSV.

## Introduction

Severe fever with thrombocytopenia syndrome (SFTS) is an emerging infectious disease caused by SFTS bunyavirus (SFTSV) [1]. This virus was originally identified in 6 provinces of central and northeastern China [1]. Later on, 10 more provinces have been added to the list of endemic regions [2]. Moreover, similar viruses have recently been found to circulate in the United States, South Korea and Japan [3, 4, 5], indicating the genus phlebovirus worldwide distribution. Although most human cases in China are sporadic, SFTS constitutes a threat to public health in China because of its epidemic potential, high fatalities (10–16%), potential for family cluster or nosocomial outbreaks by means of direct infectious blood or secretion contact, and the difficulties in treatment and prevention [6–10].

Ticks have been implicated as the primary host vector for SFTSV based on several lines of evidence. First, most of the index patients had histories of tick bites before illness onset [1, 10], second, the living environments of the patients were heavily infested by ticks [11], third, several research groups have detected SFTSV-specific nucleotide sequences, or isolated virus from ticks collected from animals, or the environment [12–14], fourth, spatial and temporal distributions of human cases are consistent with the fluctuation of certain species of ticks in a given endemic area [6]. However, most of the data supporting SFTSV transmission by ticks were obtained from molecular epidemiological surveys, and the vector for SFTSV remains unsettled.

In order to explore the role of ticks in the natural cycle of this virus, an experimental as well as a natural infection studies were conducted. Goats were chosen as the study subjects based on the following evidence: 1) goats graze on meadow all year round with a potential for close contact to ticks, and they are usually infested with ticks during spring and summer, correlating with the seasonal distribution of human cases [6]; 2) several studies have shown a high SFTSV-specific sero-prevalence in goats in the endemic regions ranging from 12 to 80% [11, 15–17], indicating goats’ high exposure to the virus under the natural environment. In this study, we detected the viral RNA in both ticks and goats, observed the sero-conversion in goats, and isolated 2 viral strains with the same identity, providing new evidence to support a relationship between ticks and their naïve susceptible hosts in SFTSV transmission.

## Materials and Methods

### Ethics and Bio-safety Statements

This study protocol was approved by the animal care committee of Jiangsu Provincial Center for Disease Prevention and Control (JSCDC) with permit number 2011–015. This protocol was designed in accordance with the experimental animal management regulation of People’s Republic of China [18]. All efforts were made to minimize suffering of animals. According to the guideline for prevention and control of SFTS issued by China Ministry of Health [19], and the written approval from JSCDC, the operation was performed under bio-safety level-2 containment conditions.

### Virus

The strain of SFTSV used in this study, Jiangsu-014, was originally isolated from a patient in 2010 in Jiangsu Province. It was propagated at 37°C in Vero cells at a multiplicity of infection (m.o.i.) of 1.0 and cultivated for 10 days. Supernatants containing viral particles were harvested, aliquoted and stored at -70°C until use. The plague-forming unit technique was used to measure the virus titer on Vero cells.

### Experimental Infection

Experimental infection study was conducted in a bio-safety level-2 laboratory in JSCDC [19]. A cohort of ten 6-month-old male white goats (Yangtze river delta) was imported from SFTS-free region, and no-previous SFTSV exposure was confirmed by lack of detection of viral RNA and antibodies from their sera as described previously[15, 20]. Of the 10 goats, 5 were subcutaneously inoculated with doses calculated to contain 10^7^ plague-forming units (p.f.u.) of virus in a 3-ml volume respectively, and the other 5 were injected with equal volume of phosphate buffer solution (PBS). After inoculation, all animals were reared together, and monitored daily for clinical symptoms. At a set time point (9:00 am) from Day 1 to Day 8 after infection, a daily serum (1ml), nasopharyngeal and anal swab samples (2ml) were separately taken from each animal in the cohort, and the samples were subjected to RNA extraction by using RNeasy mini kit (Qiagen). TaqMan quantitative real-time RT-PCR was performed as previously described [20]. For antibody detection, a double-antigen sandwich ELISA kit (Xinlianxin Bio-Tech, China) was used for all serum samples to test total antibodies (IgG and IgM) against SFTSV [15].

### Natural Infection Site Description

**Fig 1:**
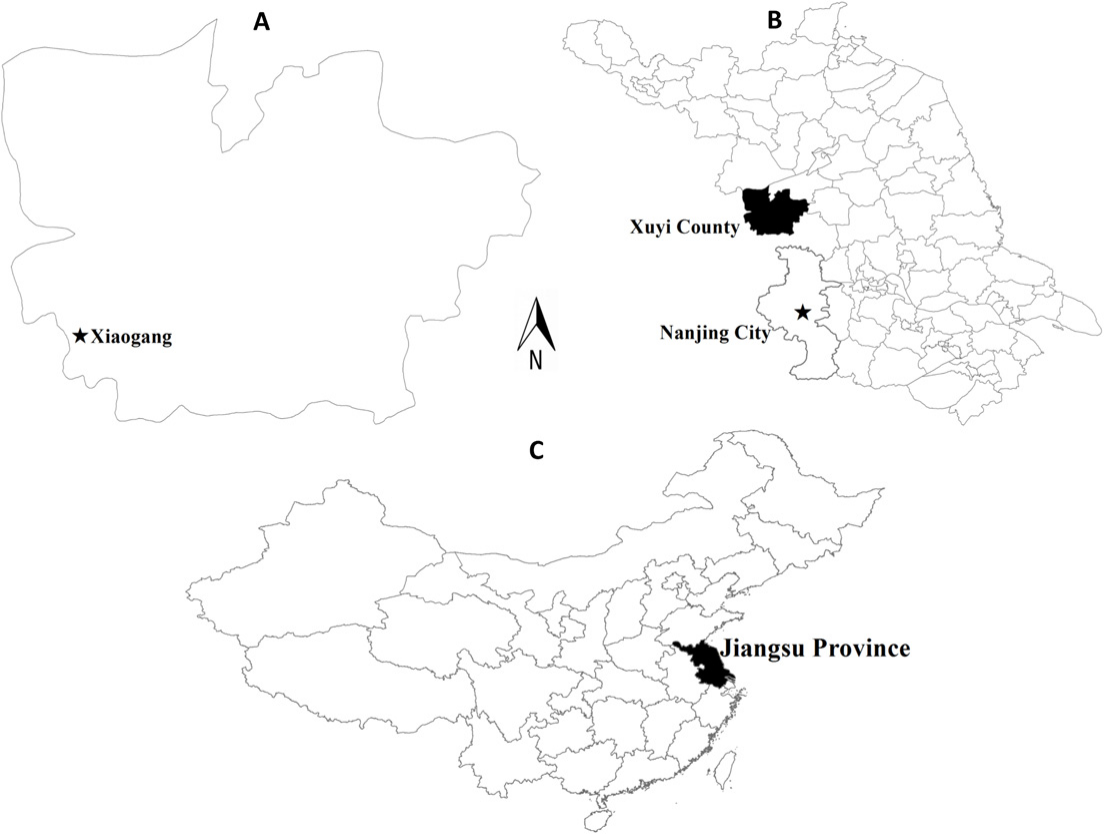
Location of the natural infection study site, Xiaogang village (indicated with a star in 1A) in Xuyi County, Jiangsu Province, China. Maps 1B and 1C show location of Xuyi County in Jiangsu Province and Jiangsu Province in China, respectively.

The natural infection study site called Xiaogang is a natural village administered by Xuyi County, Jiangsu Province. It is located in the north-west of province capital, Nanjing and in the boundary between Jiangsu and Anhui Provinces, with a latitude of 33°N, and a longitude of 118.05°E, respectively (Fig 1). This village consists of about 20 hills with an average altitude of 150m above the sea level. The subtropical-temperate monsoon climate provides a mean annual temperature of 14.7°C and an average annual precipitation of 1005.4mm. The well-developed herb and patchy shrub flora constitute the local plant community. Most of the residents in this village are farmers, mainly involved in domestic animal herding, wild herbs collecting and crop planting. The domestic animals, including goats and cattle, are severely infested by ticks in spring and summer. A recent serological survey in this area demonstrated a SFTSV infection prevalence of 3.06% for humans and 34.80% for animals (goat, cattle, pig, chicken, goose), respectively [16].

### Natural Infection

This natural infection study started from 19 April, 2012. Local temperature and relative humidity were 19–25°C and 60–65%, respectively. The vegetation was completely restored, and questing ticks were found on the grass in the open habitat of the study site. Similar to the experimental infection, a cohort of five naïve 6-month-old male white goats (Yangtze river delta) was imported from SFTS-free region, and each individual was numbered by installation of ear tag. The goats were allowed to graze on meadows freely in the daytime from 8:00am to 5:00pm, and held in stable at night. To monitor new infection of SFTSV, a daily serum sample (1ml) and a parasitic tick sample (up to ten ticks collected from host skin) were taken from each animal. All the samples were stored in 1.5-ml micro-centrifuge tubes at -20°C and immediately sent to laboratory for testing. For serum samples, SFTSV specific RNA and antibody were detected by the methods described above. Collected parasitic ticks from each animal were sorted as described previously [21], ground up by a mixer mill (Tissue Lyser LT, Qiagen, USA) according to the manufacturer’s instructions. After a brief centrifugation, the supernatant of lysate (300μl) was used for RNA extraction (RNeasy mini kit, Qiagen), which would be used as template for viral RNA amplification by real-time RT-PCR [20]. The natural infection study was terminated when all goats sero-converted.

### Collection of Free-Living Ticks and Mosquitoes for SFTSV RNA Detection

During the natural infection study period, two dominant arthropods in the area, the free-living ticks and mosquitoes, were collected as described by Schwarz [22] and Turell [23], respectively, from the grassland where goats grazed. All the samples were subjected to SFTSV RNA detection as described above.

### Virus Isolation and Sequence Analysis

For all the collected samples, including goat sera, ticks, and mosquitoes, once the RT-PCR proved positive, the corresponding sample was used for virus isolation [1]. The isolated virus was sequenced and subjected to phylogenetic analysis by using MEGA 5.05 software and compared with the published SFTSV strains sequences.

## Results

### Experimental Infection

**Fig 2:**
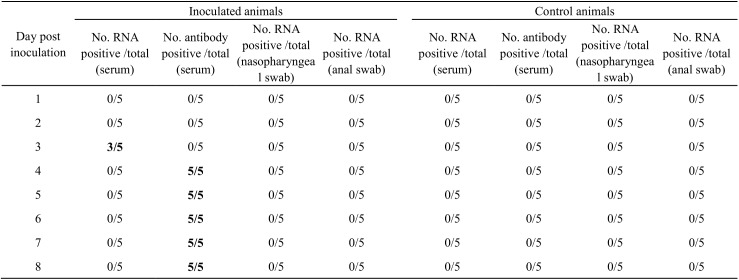
SFTSV RNA and specific antibodies detected in goats in experimental infection study.

After challenge with SFTSV, only 3 of 5 goats showed a transient viremia on Day 3 post-infection which lasted for less than 24 hours long (Fig 2). Antibodies became demonstrable on all inoculated animals from Day 4 (Fig 2). No viral RNA was detected from either nasopharyngeal or anal swab samples in the every inoculated animal (Fig 2), suggesting that infected animals did not shed virus to the outside through respiratory or digestive tract route. For the control animals, no viral RNA was detected from either swab or serum samples, consistent with a lack of antibody response over the whole testing period (Fig 2), demonstrating no evidence for virus transmission between animals by close contact mode. No visible clinical signs of infection were observed in the cohort of goats over the testing period.

### Goat Natural Infection

**Fig 3:**
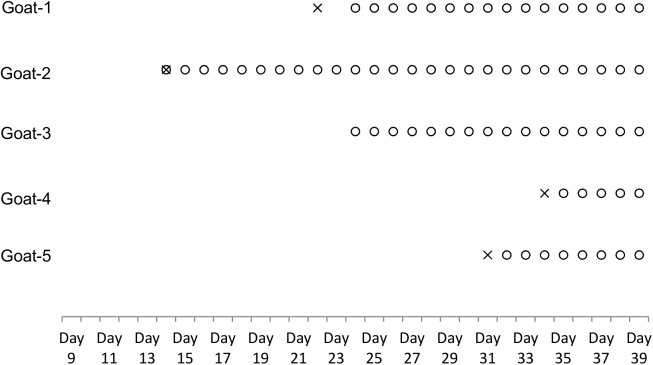
Time course of SFTSV-specific RNA and antibody response in goats in natural infection study. 5 naïve goats were farmed in SFTSV endemic site from Day 0 (19 April, 2012), and daily serum samples were taken from each goat to monitor viral infection. 4 of 5 goats in the cohort were viremic over a very short period (less than 24hr) after infection (from Day 14 to Day 34), soon occupied by a timely mounting antibody response simultaneously or 1–2 days later, which effectively controlled the infection. × viral RNA detected in goats sera; ○ viral total antibodies detected in goats sera.

The goats were observed to be gradually infested by ticks when they were farmed in the study site from Day 0 (April 19, 2012). After 13 days of incubation, the first goat (goat-2) was found to be infected on Day 14 as both viral-specific RNA and antibody from a serum sample were detected (Fig 3). Then the viral RNA and sero-conversion were sequentially observed in other animals studied, and the last goat’s (goat-4) infection was on Day 34 (Fig 3). Similar to the result of experimental infection, the goats were viremic over a very short period (less than 24hr) after viral infection, soon occupied by a timely mounting antibody response which effectively controlled the infection. The whole cohort did not show any specific clinical signs of illness, and all survived infection. The fact that all the goats got infected in a short period of time (within 21days, from Day 14 to Day 34) indicates that the pathogen density is high in the local site.

### Viral Detection in Ticks and Mosquitoes

**Fig 4:**
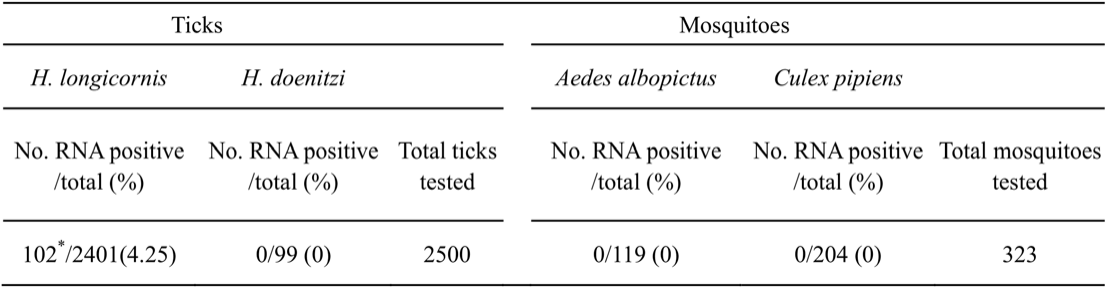
SFTSV viral RNA detected in ticks and mosquitoes in natural infection study. * 102 *H*. *longicornis* ticks included in 12 tick samples were viral RNA positive.

**Fig 5:**
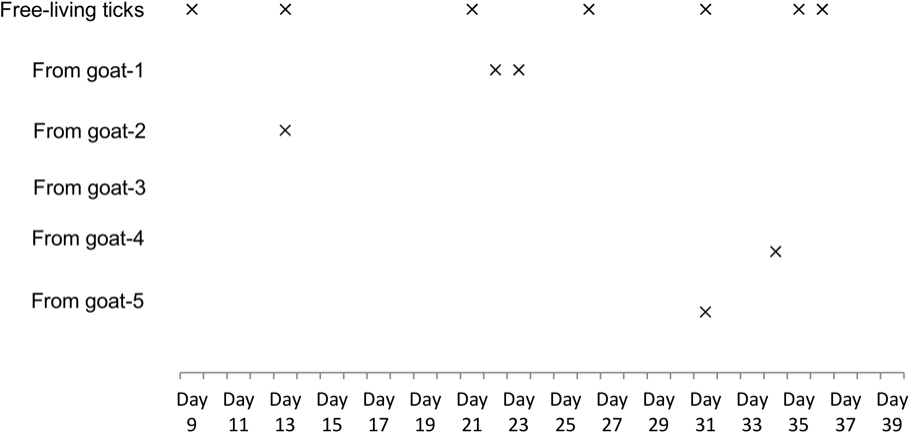
SFTSV RNA detection from *H*. *longicornis* ticks in natural infection study. The free-living and parasitic tick samples were collected from natural environment and animal cohort including 5 goats (called from goat-1, -2, -3, -4, -5), respectively, during the period Day 0-Day 39. All the samples were subjected to SFTSV RNA detection as described in Materials and Methods. × RNA detected from tick samples.

Of the 2500 ticks collected from natural environment and goats in the study site, only two species, *Haemaphysalis longicornis* (*H*. *longicornis*) and *Haemaphysalis doenitzi* (*H*. *doenitzi*) were identified with *H*. *longicornis* being the dominant one (96.04%, 2401/2500) (Fig 4). Viral RNA was detected from *H*. *longicornis*, but not *H*. *doenitzi* (Fig 4). 12 samples including 102 *H*. *longicornis* ticks (4.25%, 102/2401) were viral RNA positive (Figs 4 and 5). The time that the viral RNA was first detected from free-living *H*. *longicornis* was Day 9 (Fig 5), 5 days earlier than that from the first infected goat (Day 14) (Fig 3). Additionally, the viral RNA positivity of parasitic ticks collected from goat-1, -2, -4, and -5 was temporally close to the time point when the hosts were in their transient viremic phases (Figs 3 and 5), suggesting an effective clearance of circulating virus by serum antibody. No viral RNA was detected from mosquitoes (323 in total, including *Aedes albopictus* and *Culex pipiens* species) throughout this study (Fig 4).

### Virus Isolation and Sequence Analysis

**Fig 6:**
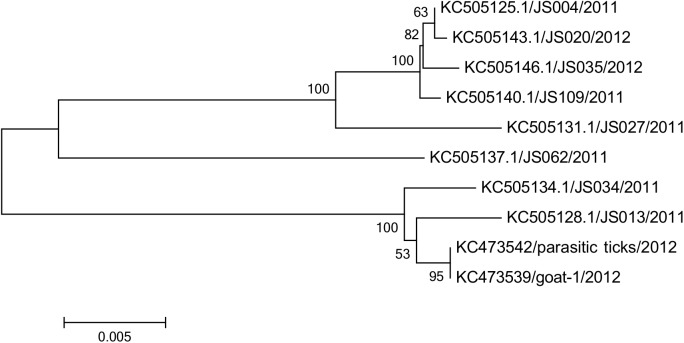
Phylogenetic analysis of genetic small segments of severe fever with thrombocytopenia syndrome virus (SFTSV) isolated from one infected goat and its parasitic ticks (*H*. *longicornis*) sample in this study, and from SFTS patients in 2011–2012. The tree was constructed by using the neighbor-joining method with MEGA 5.05. Scale bar indicates nucleotide substitutions per site.

Only two isolates were obtained from one infected goat (goat-1) serum and its parasitic tick (*H*. *longicornis*) sample, respectively, collected at the same day (Day 22), although virus isolation was attempted on all viral RNA positive samples. Phylogenetic analysis of the S segment of two SFTSV isolates in this study is genetically close to the 8 SFTS patient-derived isolates in 2011–2012 from Jiangsu Province by sharing more than 95% identity (Fig 6).

## Discussion

In this study, an experimental as well as a natural infection studies in goats were conducted to investigate the role of ticks in the natural cycle of SFTSV. In the natural infection study, we were able to detect the viral RNA in ticks and goats, observe sero-conversion in goats, and also isolate two virus strains from one infected goat serum and one of its parasitic tick samples, respectively, which share a high sequence homology. These findings help to understand the virus transmission mechanism in the natural settings.

Unlike human infections, which can cause overt clinical manifestation, and transmit virus by means of human-to-human contact [6], the goats inoculated with SFTSV exhibited no signs of disease, didn’t shed virus to the outside through respiratory or digestive tract route as described in the experimental infection study, suggesting that the viral transmission cycle could not be established effectively without certain species of arthropods as vehicle in the natural settings. In the natural infection study, the results that the naïve animals were infected after ticks’ infestation and two recovered viral isolates shared high sequence homology, demonstrate that etiologic agent for goat cohort’s natural infection comes from environmental factors. Of the two dominant arthropods in the local site, viral RNA was only detected in ticks rather than mosquitoes. Based on all these findings, we speculate that ticks, especially the predominant species, *H*. *longicornis*, act as SFTSV vector, and transmit the virus to naïve goats. However, we could not rule out the possibility that other arthropods besides ticks, e.g. fleas and sandflies, may also be enzootic SFTSV vectors, considering the fact that our natural infection study was carried out in an open environment.

The existence of SFTSV in ticks and the transmission between ticks and goats indicate that SFTS might well be a zoonotic disease, although the viral reservoir remains to be discovered. Conceivably, SFTSV is maintained in an enzootic transmission cycle among ticks and wild animals. Any accidental virus “spillover” from this cycle by ticks bite may generate infection or outbreak in humans. Ticks are obligate blood-feeders that require an animal host to survive and reproduce. Although some species of ticks feed on specific host animals, some have a wide range of hosts and transmit many human or animal pathogens [24]. In this study, of the two identified tick species, only *H*. *longicornis* was found to carry and transmit SFTSV, but we cannot exclude *H*. *doenitzi*’s possible role as SFTSV vector. Since *H*. *doenitzi* accounted for only 4% of all the ticks collected, more samples are needed for the assessment of the competence of this tick species in virus transmission.

Similar to other vector-borne diseases [25], the increase in human infection of SFTSV in China is mainly caused by anthropogenic interventions. In China, farmers are recommended by local governments to breed domestic animals, such as sheep, goats, and cattle for economic purposes. The husbandry of these free-ranging animals in SFTSV endemic areas has dramatically promoted ticks population growth and virus expansion [16]. Additionally, ruminant trade may also facilitate the spread of some viruses as seen in the case of the Rift Valley Fever virus in Saudi Arabia and Yemen [26], we have also found a SFTSV-specific sero-prevalence of 8%, and severe ticks infestation in a flock of goats in SFTSV-free region in China (personal communication), highlighting the threat of this virus expansion into other parts of China and world by live animal trade.

In conclusion, as an emerging pathogen circulating mainly in East Asia, with a tendency of spreading to other parts of the world, and in the absence of an effective drug or vaccine, the findings in this study may help local health authorities formulate and focus preventive measures to contain SFTSV infection.
